# Identification of prognostic biomarkers for glioblastomas using protein expression profiling

**DOI:** 10.3892/ijo.2011.1302

**Published:** 2011-12-15

**Authors:** YONG JUNG, KYEUNG MIN JOO, DONG HO SEONG, YOON-LA CHOI, DOO-SIK KONG, YONGHYUN KIM, MI HYUN KIM, JUYOUN JIN, YEON-LIM SUH, HO JUN SEOL, CHUL SOO SHIN, JUNG-IL LEE, JONG-HYUN KIM, SANG YONG SONG, DO-HYUN NAM

**Affiliations:** 1 Department of Neurosurgery, Samsung Medical Center and Samsung Biomedical Research Institute, Sungkyunkwan University School of Medicine, Gangnam-Gu, Seoul 135-710; 2 Cancer Stem Cell Research Center, Samsung Medical Center and Samsung Biomedical Research Institute, Sungkyunkwan University School of Medicine, Gangnam-Gu, Seoul 135-710; 3 Department of Pathology, Samsung Medical Center and Samsung Biomedical Research Institute, Sungkyunkwan University School of Medicine, Gangnam-Gu, Seoul 135-710; 4 Department of Anatomy, Seoul National University College of Medicine, Chongno-Gu, Seoul 110-799; 5 Department of Biotechnology, Yonsei University, Seoul 120-749, Republic of Korea

**Keywords:** biomarker, therapeutic target, glioblastoma, tissue micro-array, bioinformatics, automated image analysis

## Abstract

A set of proteins reflecting the prognosis of patients have clinical significance since they could be utilized as predictive biomarkers and/or potential therapeutic targets. With the aim of finding novel diagnostic and prognostic markers for glioblastoma (GBM), a tissue microarray (TMA) library consisting of 62 GBMs and 28 GBM-associated normal spots was constructed. Immunohistochemistry against 78 GBM-associated proteins was performed. Expression levels of each protein for each patient were analyzed using an image analysis program and converted to H-score [summation of the intensity grade of staining (0–3) multiplied by the percentage of positive cells corresponding to each grade]. Based on H-score and hierarchical clustering methods, we divided the GBMs into two groups (n=19 and 37) that had significantly different survival lengths (p<0.05). In the two groups, expression of nine proteins (survivin, cyclin E, DCC, TGF-β, CDC25B, histone H1, p-EGFR, p-VEGFR2/3, p16) was significantly changed (q<0.05). Prognosis-predicting potential of these proteins were validated with another independent library of 82 GBM TMAs and a public GBM DNA microarray dataset. In addition, we determined 32 aberrant or mislocalized subcellular protein expression patterns in GBMs compared with relatively normal brain tissues, which could be useful for diagnostic biomarkers of GBM. We therefore suggest that these proteins can be used as predictive biomarkers and/or potential therapeutic targets for GBM.

## Introduction

Glioblastomas (GBMs), the most common primary brain tumor in adults, show a median survival of <12 months due to its resistance to current medical treatments. Several genetic aberrations have been shown in GBMs (1). However, the molecular markers that correlate with the clinical outcome of GBMs are still required to establish the comprehensive molecular fingerprint. Such molecular profiling may eventually lead to diagnostic biomarkers and/or targeted therapeutic approaches that can improve the clinical outcome (2).

The Cancer Genome Atlas Research Network provided an integrative analysis of mRNA and DNA data (3). The landmark study was of particular importance because they integrated a large number of datasets and presented an unbiased and systematic cancer genome analysis (4). Yet, what is still lacking is a robust protein and clinical dataset, which could provide functional level understanding and thereby complement genomic and transcriptomic data. Therefore, we applied the tissue microarray (TMA) analysis to acquire the protein expression data (5,6).

Previous reports demonstrated brain tumor profiling using TMA is feasible (2,7–10). However, the conventional profiling has inherent limitations because they only provide categorical values. Moreover, acquisition of TMA data is often plagued with high subjectivity originating from intra- and inter-observer variations (11). Now that image analysis technique has improved, it would benefit us to use it to reduce systematic errors and human bias (12,13). Therefore, here, we used image analysis tool to more objectively quantify the values.

We applied the TMA technology for an exhaustive immunoprofiling of GBMs with integrating clinical variables in an attempt to identify new biomarkers for diagnostic and/or prognostic utility.

## Materials and methods

### Patients and tissue collection

Sixty-two GBM samples were obtained at Samsung Medical Center (Seoul, Korea) with written informed consent in accordance with the institutional review board between January 2004 and December 2006 (original set). Patients were managed according to established diagnostic and therapeutic protocols, including surgical resection and subsequent chemoradiotherapy. A macroscopic total resection was performed in 47 of 62 patients (75.8%), a partial resection in 14 of 62 patients (22.6%), and a biopsy only in one of 62 patients (1.6%). Tumor samples were re-evaluated by neuropathologists to confirm the diagnosis according to World Health Organization criteria. All patients underwent subsequent radiotherapy (60 Gy in 2 Gy fractions) after surgical resection. Fifty of 62 patients received temozolomide concurrent chemoradiotherapy with a median of 4 cycles (range, 1–9 cycles). However, the 12 others (20%) did not receive chemotherapy because of clinical deterioration during radiotherapy. There was no follow-up loss.

Another independent set consisting of 82 GBM patients diagnosed between January 2004 and December 2007 from the same institute was used for validation (validation set). The 82 GBMs of the validation set were also managed according to established diagnostic and therapeutic protocols. A macroscopic total resection was performed in 59 of 82 patients (72.0%), a partial resection in 19 of 82 patients (23.2%), and a biopsy in 4 of 82 patients (4.9%). All patients underwent subsequent radiotherapy after surgical resection. Fifty of 82 patients had temozolomide concurrent chemoradiotherapy with a median of 5 cycles (range, 1–17 cycles). There were 6 follow-up losses.

Of the 82 patients, 39 were included in both the original and validation datasets owing to the difficulty of acquiring adequate number of surgical samples or public TMA data of GBMs. Instead, the surgical samples of the 39 GBMs were newly processed for TMA and expression of proteins was re-examined. Detailed patients’ clinical data of the original dataset was also reported in Kong *et al* (14).

### Construction of tissue microarray

Surgical samples were fixed in 10% formalin solution (Sigma-Aldrich) for 24 h at 4°C within 24 h after surgery and then paraffin-embedded. A representative area of each GBM was marked on an H&E section of each patient’s paraffin block avoiding necrosis and extensively vascularized area. Corresponding tissue core of 2 mm diameter was extracted from the original donor block using an arraying machine (MTA-1, Beecher Instruments). The cores were fit into a vertical hole that was bored in a recipient paraffin block. Recipient blocks were incubated at 58°C for 5 min, pressed on a hot plate for 3 min, and cooled in ice water to enable tissue cores to integrate into the recipient block. Sections of 4 μm thickness were cut from each array block.

### Selection of biomarkers

Recently, frequent genetic alterations of GBM in three critical signaling pathways were reported by the Cancer Genome Atlas (3). Accordingly, expressions of i) receptor tyrosine kinase (RTK)-RAS-PI(3)K pathway proteins (X05, X10, X12, X38, X61, X63, X64, X66, X67, X68, X69, X70, X71, X76, X77, X78, X82, X83), ii) p53 signaling proteins (X28, X35, X36), iii) RB signaling proteins (X08, X14, X15, X31, X32, X33, X34, X37, X39, X50) were selected (Table I). Since the profound characteristics of GBM include invasion of tumor cells and proliferation of endothelial cells, proteins promoting cell invasion (X43, X44, X45, X79, X80) and angiogenesis related proteins (X46, X47, X51, X54, X60, X72, X73, X74, X75) were also examined (Table I). DNA and histone modification (X01, X17, X18, X19, X20, X24, X25) and neural stem cell markers (X06, X07, X21, X40, X49, X52, X53, X56, X57, X58, X65, X84) were included regarding that cancer stem cells of GBM which may be related with radio- and/or chemo-resistance, could share molecular characteristics with neural stem cells and have alteration in the DNA and histone methylation and/or acetylation (Table I). Several common oncogene (X02, X04, X09, X11, X16, X22, X23, X27, X42) and apoptosis regulators (X03, X13, X41, X55, X81) were also analyzed. Phosphorylation-specific antibodies against p70 S6 kinase (X61), Akt (X63), PDGFR-α (X68), PDGFR-β (X71), VEGFR2 (X74), VEGFR2/3 (X75), EGFR (X82) were included since they are the most important targets of newly-developing targeting agents (Table I).

### Immunohistochemistry and generation of protein expression values

Immunohistochemical staining against 78 tumor-associated gene products was done using a standard procedure (14). Briefly, sections of TMAs were prepared on the slide, baked at 55°C for 30 min, deparaffinized in xylene and rehydrated in graded concentrations of ethanol. Antigens were retrieved in 10 mM citrate buffer (pH 6.0, Dako) for 5 min in MicroMED T/T Mega microwave (Milestone). Endogenous peroxidase activity was blocked by incubation in 0.3% hydrogen peroxide in methanol. Primary antibodies (overnight at 4°C, Table I), biotinylated secondary antibodies (1:200, 1 h at room temperature, Vector) and an ABC kit (1 h at room temperature, Vector) were applied sequentially. Diaminobenzidine tetrahydrochloride (DAB) was used as the enzyme substrate. Specificity of primary antibodies was validated by i) no immunoreactivity in GBM sections which were reacted without primary antiserum as negative controls, and ii) comparing staining pattern of each antibody with previous studies that used the same antibody.

One hundred and eight subcellular location-specific proteins were extracted based on previously reported cellular localizations of 78 proteins (e.g., Myc_Cytoplasm). Immunostained TMA slides were scanned by Aperio Scan Scope CS System and converted into image files. Staining intensity of each protein (grade 0, negative; 1, weak but detectable above control; 2, moderate; and 3, strong) and percentage of positively stained cells for each intensity were automatically analyzed in the whole area of each spot by TissueMine (Bioimagene Co.) (15).

TissueMine determines positive versus negative staining based on the colorimetric differences between stained and unstained subcellular location (i.e. nuclei; positive nuclei demonstrating brown staining consisted of pixels with red greater than blue). These algorithms then used the gray scale (0–255) to quantitate intensity of staining. The grade of intensity is determined by percentage of positive cells. For example, the grade 1 means that the percentage of positive cells is between 10–25%.

**Figure 1:**
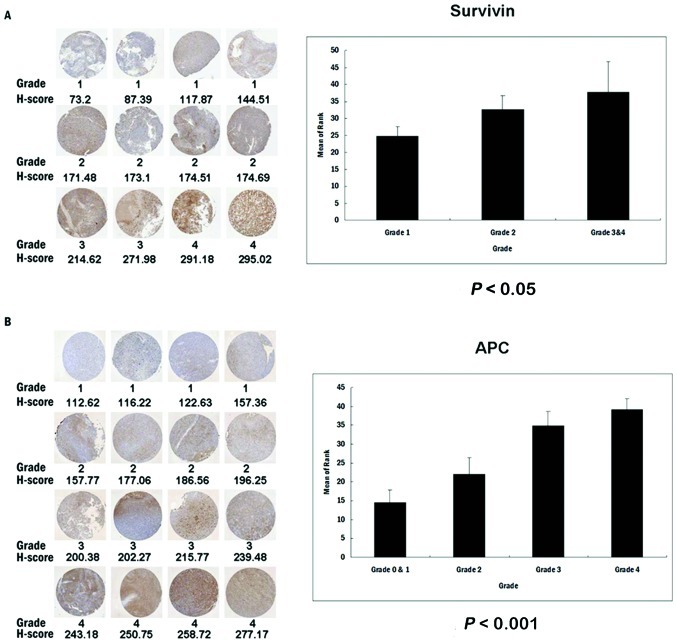
Comparison of scoring methods between manual grade and H-score from image analysis result. Representative expressions of survivin (A) and APC (B) proteins studied by IHC on TMAs and their grade score and H-score. Magnification, x50. In the right column, plots show that grades of spots and their rank values of H-score have high correlation. From Kruskal-Wallis test, statistically significant differences of three or four groups can be determined. For survivin: p<0.05; for APC: p<0.001.

For each observed tissue component, a summary value we refer to as H-Score was calculated. This consists of a sum of the percentages of positively stained cells multiplied by a weighted intensity of staining:

$$\text{H}-\text{score}=\sum_{\text{i}=0}^{3}{\text{P}}_{i}*i$$

where P*_i_* is the percentage of stained cells in each intensity category, and *i* is the intensity for *i* = 0, 1, 2, 3 (16–20). Kruskal-Wallis test and plotting were performed to verify the utility of H-score and to confirm the correlation of it with the manual grade. The validity of this automated process was confirmed by clinical experts (Fig. 1).

### Data analysis

#### Exclusion criteria and normalization

We excluded samples according to the exclusion criteria: i) GBMs missing >15% of the protein expression values, ii) recurrent GBMs, iii) GBMs without clinical data. To remove non-biological origin of variation between arrays, we performed the quantile-normalization on the assumption that the distribution of protein expressions for each patient would be the same, similar to what is already observed for patient’s gene expression distribution (21). In the quantile-normalization, a qqplot was utilized to compare distribution of datasets from patients. Projecting each value onto the unit diagonal in the qqplot makes distributions identical. To adapt the method for TMA data, we transposed TMA data matrix to set columns and rows to samples and proteins and used normalize.quantiles function in affy package of R program.

#### Hierarchical clustering and survival analysis

Hierarchical clustering (distance, standard Euclidean distance; criterion, complete-link) was performed using the R program package (22). With clustering results, we performed Kaplan-Meier survival analysis and log-rank test to compare the clinical outcomes. The influence of possible compounding factors was assessed by Cox proportional hazard models.

#### Selection of biomarker candidates

Two clusters differentiated by the hierarchical clustering from the original dataset were compared by Student’s t-test which is then used for finding differentially expressed proteins. We performed multiple testing corrections using Benjamini & Hochberg false discovery rate to correct for the occurrence of false positives (23).

#### Supervised analysis using the classification methods

We used supervised methods [support vector machine (SVM), random forest (RF), and general linear model (GLM)] to confirm the utility of whether the set of biomarker protein candidates distinguished the groups appropriately into survival status. Among the 56 cases, two-thirds of the cases from each group were randomly assigned to the training set, and the remaining one-third was assigned to the test set. A receiver operating characteristic (ROC) curve was constructed, and the area under the curve (AUC) was calculated. The class, defined as the prognosis outcome for any patient’s protein expression data using the output computed by the model, was predicted by R program package (22).

#### Validation of the biomarkers

We validated our ten prognostic markers against another independent GBM TMA dataset. Same preprocessing works (immunohistochemistry, generation of protein expression values, normalization, hierarchical cluster, survival analysis) were performed. We re-evaluated the 10 biomarkers using a publically available DNA microarray data [GSE4271 from the gene expression omnibus (24,25)]. We normalized the gene expression CEL files using Robust Multichip Averaging procedure, and PM-MM difference model was used to obtain the expression values. With nine candidate genes which corresponded to our ten prognostic markers, we calculated the Euclidean distance between the patients and constructed a corresponding distance matrix. The resulting nine-dimensional data was rescaled to a two-dimensional map using the multidimensional scaling (MDS) method. We performed the k-means clustering on the MDS result with the number of clusters set to two (k=2) to correspond to the clustering performed in TMA analysis. Survivals of the two groups were compared by the Kaplan-Meier survival analysis and log-rank test.

## Results

### Expression profiling of 108 subcellular location-specific proteins

The expressions of 78 proteins (Table I) were studied by immunohistochemistry on TMAs containing 62 GBM and 28 tumor-associated normal (normal brain tissue extracted around tumor tissue) spots proteins. For each protein, grade value (0–4) of each spot was made by pathological reading. H-scores (a sum of the percentages of positively stained cells multiplied by a weighted intensity of staining: grade 0, negative; 1, weak but detectable above control; 2, moderate; and 3, strong) were also generated by an image analysis program (12). In the generation of H-score, each protein was read in each subcellular localization, i.e., nucleus, cytoplasm and membrane, based on previously reported cellular localizations of 78 proteins (e.g., Myc_Cytoplasm). Consequently, we obtained 108 subcellular location-specific proteins (Table I). According to criteria for exclusion, 56 tumor spots and 26 normal spots were included in further analysis (male, 34; female, 22).

To verify the utility of the H-score, we compared manually constructed numerical values against automatically generated H-scores in 20 randomly selected proteins. We concluded that using H-score was reasonable for profiling protein expressions as those values were well-correlated (p<0.05, Kruskal-Wallis test, Fig. 1).

### Hierarchical clustering and survival analysis

**Figure 2:**
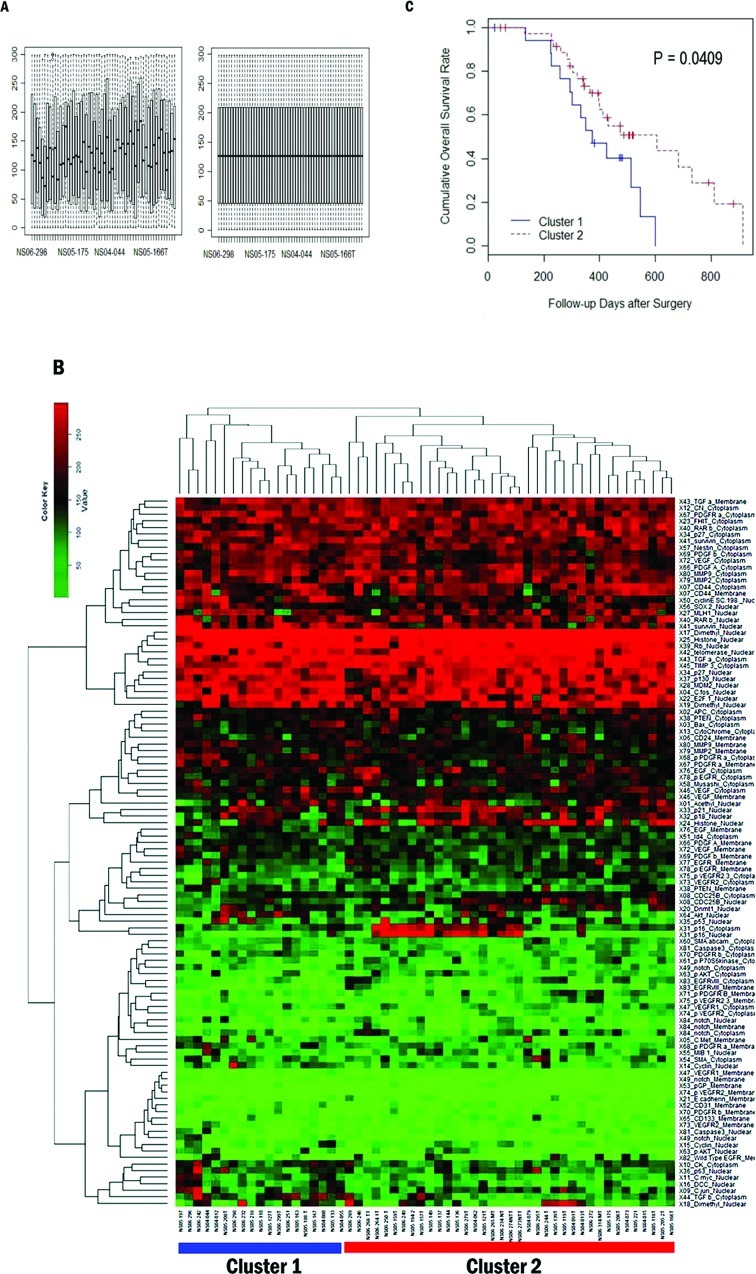
Classification of 56 patients by prognosis based on 108 protein expression values. (A) Boxplot of protein expression values before (left) and after (right) quantile normalization. The plot shows the quantile normalized distribution of protein expression values for each patient. Horizontal axis represents individual patients. Vertical axis represents H-score. (B) Heatmap and dendrogram as a result of hierarchical clustering of GBM samples. Top dendrogram represents clustering of patients. Left dendrogram represents clustering of proteins. Of two patient branches, samples in the left branch represent cluster 1 and samples in the right branch represent cluster 2, consisting of 19 and 37 patients, respectively. (C) Univariate survival analysis of overall survival by Kaplan-Meier method. Kaplan-Meier survival plot of the two clusters of patients defined by the hierarchical clustering. Cluster 1 is the poor survival group. The log-rank test shows that the difference between two curves is significant (p<0.05).

The H-scores were normalized with quantile normalization to make the data statistically comparable. We clustered the normalized data with hierarchical clustering method. Tumor and relatively-normal spots were well-divided (p=9.9904×10^−16^, Fisher’s exact test), confirming that this method was suitable for separating spots into groups having characteristic protein expressions. Using only tumor spots, we performed quantile normalization (Fig. 2A) and clustering analysis. The combined protein expression patterns defined 56 GBMs into cluster 1 (n=19) and cluster 2 (n=37) (Fig. 2B).

We performed survival analysis to determine whether the two clusters represent distinct prognostic subgroups. Indeed, cluster 2 had a significantly better survival than cluster 1 [median survival (months) of cluster 1, 11.7 (range: 0.7–20); cluster 2, 13.2 (range: 1.4–30.4), p<0.05, log-rank test, Fig. 2C]. The clinical characteristics of the two groups showed no significant difference (Table II). On multivariate survival analysis, molecular classification (clusters 1 and 2) and age (<70 and ≥70 years) were the most significant factors to distinguish patients by prognosis (p<0.01 and p<0.01, respectively, Table III). Based on these significant results, we determined that two groups from clustering analysis were clinically distinct.

### Biomarker candidates and supervised analysis

**Figure 3:**
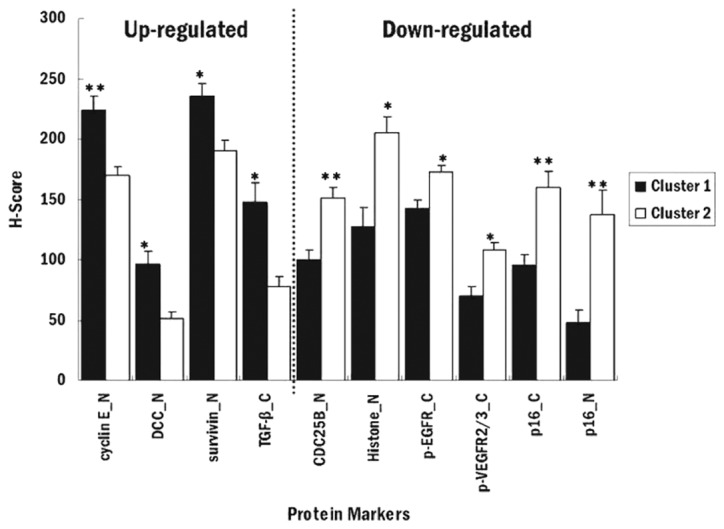
Plot for means of protein marker expressions of two groups. The comparison of means of statistically significant biomarker expression values. Mean values for good prognosis group are represented as white bar, and mean values for poor prognosis group are represented as black bar (^**^q<0.01; ^*^q<0.05, t-test).

Student’s t-tests were utilized to identify proteins whose expression significantly differed between the two groups with Benjamini & Hochberg False Discovery Rate. Ten subcellular location-specific proteins were identified (Table IV, q<0.05, CDC25B_nuclear, cyclinE_nuclear, p16_nuclear, p16_cytoplasm, TGF-β_cytoplasm, histone_nuclear, p.VEGFR2.3_cytoplasm, DCC_nuclear, survivin_nuclear, p.EGFR_cytoplasm). Of these, four proteins were overexpressed while the remaining six were underexpressed in cluster 1 (Fig. 3). With a more stringent statistical criterion (q<0.01), CDC25B_nuclear, cyclinE_nuclear, p16_nuclear and p16_cytoplasm were significantly different. Functional categorization of these proteins using the PANTHER (26) ontology database showed that they are significantly related with p53 pathway, angiogenesis, cell cycle, Ras pathway and VEGF signaling pathway (p<0.05).

Three supervised (classification) models (SVM, RF and GLM) were used to confirm that these proteins were available for classification for prognosis of GBMs. SVM shows the best performance in our data. The prediction accuracy of SVM for patient survival was 87.5% with 0.05 adjusted p-value (AP) and 80.4% with 0.01 AP. The sensitivity for better prognosis group of classifier for patient survival was 100% (specificity: 63.2%) with 0.05 AP and 94.6% (specificity: 52.6%) with 0.01 AP. ROC curves for 0.05 AP and 0.01 AP showed that classification model using 0.05 AP performs better than 0.01 AP (data not shown).

Interestingly, survivin_nuclear was the most significant factor for 0.05 AP and cyclinE_nuclear was the most significant factor for 0.01 AP for each classification model in GLM. Contrary to our expectations, the same protein was not the most important factor in both models. This discrepancy was, however, rectified when considering that these two proteins both belong to the chemo-radiation-resistant group (poorer prognosis group). In addition, further analysis by protein clustering showed that they belonged to the same cluster, implying that these two proteins had a similar pattern. Consequently, with this analysis, we could confirm that the result of hierarchical clustering, unsupervised method was well explained by supervised methods.

### Validation of selected biomarker candidates

**Figure 4:**
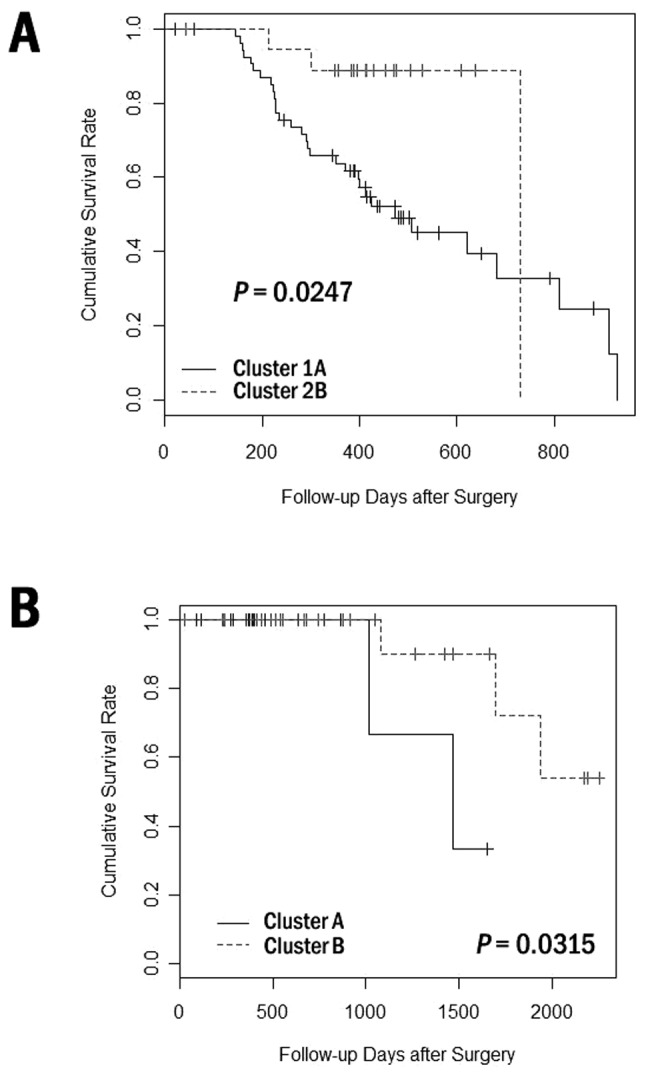
Validation of clustering and survival analysis for the 82 GBM patients from another TMA dataset and from public DNA microarray data and survival analysis of 55 independent GBM patients. (A) Kaplan-Meier survival curves of the two groups from clustering analysis of another independent TMA dataset with 10 biomarkers identified. They show statistically significant difference in the overall survival by the log-rank test. (B) Kaplan-Meier survival curves for each group from clustering analysis on public DNA microarray data were compared with overall survival. Clustering analysis was performed with nine candidate genes (corresponding to proteins of ten pairs based on their cellular localization). Differences in the survival were tested for statistical significance by the log-rank test.

In order to validate the clinical significance of the 10 markers, an independent TMA consisting of 82 GBMs was analyzed. We obtained expression values of the 10 subcellular location-specific proteins and calculated H-scores. Two groups were separated (cluster 1A and 2B, n=59 and 23, respectively). These two groups also showed statistically significant difference in the survival [median survival (days) of cluster 1A, 391 (range, 42–730); cluster 2B, 415 (range, 22–929), p<0.05, log-rank test, Fig. 4A]. The clinical characteristics of the two groups showed no significant difference (Table V). These results showed that the biomarkers obtained from the original set are valid to another independent data set.

Protein expression data are arguably more useful than transcriptional data because it provides the data at the functional level of cells, and thus it is not uncommon to observe differences in the expression of proteins and mRNAs. Nevertheless, correlation between the two types of data can be very meaningful. Therefore, we used DNA microarray data to evaluate the 10 biomarkers acquired through TMA data analysis as further validation of our approach. We selected 55 GBMs from high grade gliomas from the microarray data (GSE4271) of Phillips *et al* (24), which had both expression and clinical data. k-means clustering was carried out to group GBMs using genes corresponding to the biomarkers that we selected. We obtained two clusters consisting of 30 (cluster A) and 25 GBMs (cluster B). The survival analysis showed that the prognostic difference of the two groups was significant [median survival (months) of cluster A, 13.3 (range, 2.8–55.1); cluster B, 22.2 (range, 0.7–75.1), p<0.05, log-rank test, Fig. 4B). Survivin and cyclin E were also the most significant contributors for discriminating between two prognostic groups (GLM). According to the TMA analysis, these proteins were overexpressed in the chemo-radiation-resistant group. Interestingly, the same observation was made for their corresponding genes from the DNA microarray analysis, confirming other previous reports (27,28) and validating our data. The results here show that our TMA analysis is indeed a valid approach in differentiating prognostic groups.

### Aberrant protein expressions in GBM

Aberrant or mislocalized protein expression patterns could be useful for diagnosis of GBM. Accordingly, we further compared expressions of all 234 subcellular location-specific proteins (78 proteins) of GBMs with those of relatively-normal tissues using the original dataset. H-scores of 16 subcellular location-specific proteins were decreased and 16 ones increased significantly in GBMs (data not shown). Of those, cyclin E expression in the cytoplasm was increased whereas the expression in the nuclear was decreased in GBMs. Therefore, this particular protein could be one of mislocalized candidates specific for GBMs. Previously, cyclin E was validated as a prognostic biomarker for GBM (27), which could underline the significance of cyclin E in GBMs.

## Discussion

This study shows that molecular classification of GBMs can be accomplished based on the immunohistochemical profiles of biomarkers using the TMA methods. Recent studies have also supported that clinical application of the TMA methods to the molecular classifications of various cancers (29,30). To provide a more precise data analysis, this study utilized scaled continuous protein expression values (H-score) and could find more significant differences using parametric statistic tests with continuous variables.

The overall protein expression patterns using 108 subcellular location-specific proteins defined two survival-associated clusters. However, 108 expression values were too many for practical use. To reduce the number of markers, we performed a feature selection using Student’s t-test. Ten and four subcellular location-specific proteins were selected with 0.05 and 0.01 adjusted p-value cut, respectively. Survivin_nuclear was the most significant factor for 0.05 AP and cyclinE_nuclear for 0.01 AP. Survivin is known to be an inhibitor of apoptosis that acts via a pathway independent of Bcl-2. Previous studies showed a correlation between increased protein/mRNA levels of surviving and adverse prognosis in various cancers (28,31–34). Cyclin E mediates the initiation of DNA synthesis in the late G1 phase by activating cyclin-dependent kinases 2. Abnormal expressions of cyclin E (27,35,36) and other biomarkers (37–42) we selected have frequently been found in cancer cells.

In this study, phosphorylation-specific antibodies against p70 S6 kinase, Akt, PDGFR-α, PDGFR-β, VEGFR2, VEGFR2/3 and EGFR were included since they are the most important targets of newly-developing targeting agents. In contrast to our expectation, phosphorylated EGFR and VEGFR2/3 were inversely correlated with worse prognosis. Although EGFR and VEGFR are heavily activated in GBMs, agents targeting them showed disappointing results in clinical trials (43,44). Therefore, our results could reflect those results and indicate that other specific targets that have prognostic significance need to be elucidated.

In this study, we could not obtain non-neoplastic human brains as controls. Alternatively, tumor-associated normal tissues that were adjacent to the tumors were utilized. These tissues may harbor GBM cells, therefore, we selected the outermost region of the surgical samples. In addition, the tissues were clearly separated from the tumor cores by hierarchical clustering. This result indicates that there was minimal GBM cell-contamination, and, though not the most ideal, they are valid as controls.

It could be argued that due to the immense heterogeneity of cancer specimens, the normalization method we adopted may not seem pertinent to our study. Also, the quantile-normalization has an admitted limitation in that it removes outliers which could be of meaningful value. Our H-score method scaled the range of expression of all the cells found in the entire area of each spot of TMA, therefore, it would represent the heterogeneity well. Moreover, since the scaled range cannot have outliers, we circumvented the limitations of normalization by our scoring method.

Our 10 biomarkers are proven powerful to predict prognosis of GBM by analyzing three independent datasets. Moreover, the suggested biomarkers are optimal for practical use in pathology laboratories with respect to cost and time of prognostic evaluations. They also can provide the basis for developing new personalized approach as well as drug discovery. In addition we expect that our findings at the protein level can complement the transcriptomic and genomic data of GBMs (3,45) and would improve the molecular understanding of GBM.

For clinical application of the results, further analysis with a larger sample set and more detailed validation are still necessary. Nevertheless, the approach used in this study suggests that subjective interpretation of TMA profiling can be minimized and that larger scaled TMA analysis can be simultaneously performed. To our knowledge, this is the first TMA data analysis study based on quantitative values of protein expression using an image analysis tool. By applying bioinformatics techniques with large-scale data, it is now possible to perform comprehensive analysis and identify the cluster set of protein biomarkers for GBM.

## Figures and Tables

**Table I:** The 78 antibodies used for immunohistochemical study.

Code	Antibody	Source	Dilution	Retrieval method	Localization	No. of analyzed patients	Observed expression pattern in GBMs
X01	Acetyl H3(lys9)	Cell Signaling, 9671S	1:100	Microwave	N	62	N
X02	APC	GeneTex, GTX16794	1:20	Microwave	C	62	C, M
X03	Bax	Invitrogen, 33–6400	1:100	Microwave	C	62	C
X04	c-Fos	Neomarker, RB-9413-P1	1:100	Microwave	N	62	N
X05	c-Met	Invitrogen, 370100	1:50	Microwave	M	62	C, M
X06	CD24	Lab Vision, MS-1279	1:20	Microwave	M	62	C, M
X07	CD44	Lab Vision, MS-668	1:100	Microwave	C, M	62	C, M
X08	CDC25B	Lab Vision, MS-123	1:10	Microwave	C, N	62	N
X09	c-Jun	Calbiochem, OP55	1:40	Microwave	N	62	C, N
X10	c-K-Ras	GeneTex, GTX16819	1:10	Microwave	C	62	C
X11	c-Myc	Lab Vision, MS-139	1:50	Microwave	N	62	C, N
X12	c-N-Ras	GeneTex, GTX13054	1:10	Microwave	C	62	C
X13	Cytochrome C	Lab Vision, MS-1192	1:100	Microwave	C	62	C
X14	Cyclin D1	Lab Vision, RM-9104	1:50	Microwave	N	62	N
X15	Cyclin E	Lab Vision, MS-1060	1:20	Microwave	N	62	N
X16	DCC	BD, 554223	1:100	Microwave	N	62	C
X17	Dimethyl histone H3 (lys4)	Upstate, 07–030	1:100	Microwave	N	62	N
X18	Dimethyl histone H3 (lys9)	Upstate, 07–212	1:100	Microwave	N	62	N
X19	Dimethyl histone H4 (arg3)	Upstate, 07–213	1:100	Microwave	N	62	N
X20	Dnmt1	GeneTex, GTX13537	1:200	Microwave	N	62	C, N
X21	E-cadherin	Zymed, 1379115	1:50	Microwave	M	62	C
X22	E2F-1	Lab Vision, MS-879	1:20	Microwave	N	62	C, N
X23	FHIT	Lab Vision, MS-9471	1:200	Microwave	C	62	C
X24	Histone H1 (B419)	GeneTex, GTX72121	1:50	Microwave	N	62	N
X25	Histone H3 (dimethyl K9)	Abcam, ab7312	1:100	Microwave	N	62	N
X27	MLH1	BD, 551091	1:25	Microwave	N	62	N
X28	MDM2	Lab Vision, MS-291	1:100	Microwave	N	62	N
X31	p16	BD, 550834	1:10	Microwave	C, N	62	C, N
X32	p18	Lab Vision, MS-858	1:20	Microwave	N	62	C, N
X33	p21	Lab Vision, MS-891	1:40	Microwave	N	62	C, N
X34	p27	Lab Vision, MS-256	1:40	Microwave	C, N	62	C, N
X35	p53	Lab Vision, MS-186	1:30	Microwave	N	62	N
X36	p63	Lab Vision, MS-1081	1:50	Microwave	N	62	C, N
X37	p130	Lab Vision, MS-866	1:40	Microwave	N	62	N
X38	PTEN	Lab Vision, MS-1601	1:20	Microwave	C, M	62	C, M
X39	Rb	Lab Vision, RB-1441	1:40	Microwave	N	62	N
X40	RAR-β	Lab Vision, MS-1342	1:20	Microwave	C, N	62	C, N
X41	Survivin	Lab Vision, MS-1202	1:25	Microwave	C, N	62	C, N
X42	Telomerase	Lab Vision, RB-10328	1:40	Microwave	N	62	N
X43	TGF-α	GeneTex, GTX16768	1:10	Microwave	C, M	62	C, M
X44	TGF-β	GeneTex, GTX21279	1:1000	Microwave	C	62	C, M
X45	TIMP-3	Lab Vision, RB-1541	1:10	Microwave	C	62	C, M
X46	VEGF	Lab Vision, MS-350	1:50	Microwave	C, M	62	C, M
X47	VEGFR1	Novus Biologicals, NB100–685	1:10	Microwave	C, M	62	C, M, N
X49	Notch	Cell Signaling, val 1744	1:200	Microwave	C, M, N	62	C, M, N
X50	Cyclin E (SC-198)	Santa Cruz, SC-198	1:200	Microwave	N	62	C, N
X51	CD31	Dako, M0823	1:200	Microwave	M	62	C, N
X52	Id4	Santa Cruz, SC-13047	1:100	Microwave	C	62	C, M
X53	pGP	Dako, M3521	1:50	Microwave	M	62	C, M
X54	SMA	Dako, M0851	1:100	Microwave	C	62	C, N
X55	MIB-1	Dako, M7240	1:300	Microwave	N	62	N
X56	SOX-2	R&D, MAB2018	1:50	Microwave	N	62	C, N
X57	Nestin	Abcam, ab5968	1:500	Microwave	C	62	C
X58	Musashi	Chemicon, ab5977	1:500	Microwave	C	62	C, N
X60	SMA (Abcam)	Abcam, ab5694	1:300	Microwave	C	62	C
X61	p-p70 S6 kinase (Thr421/Ser424)	Cell Signaling, 9204C, N	1:100	Microwave	C	62	C, N
X63	p-AKT (Ser473)	Cell Signaling, 9277	1:25	Microwave	C, N	62	C, N
X64	Akt	Cell Signaling, 9272	1:50	Microwave	N	62	C, N
X65	CD133	Abcam, 19898	1:200	Microwave	M	62	C, M
X66	PDGF-A	Santa Cruz, SC-128	1:100	Microwave	C, M	62	C, N
X67	PDGFR-α	Santa Cruz, SC-338	1:100	Microwave	C, M	62	C, M
X68	p-PDGFR-α (Tyr720)	Santa Cruz, SC-12910	1:100	Microwave	C, M	62	C, M
X69	PDGF-B	Santa Cruz, SC-127	1:200	Microwave	C, M	62	C
X70	PDGFR-β	Santa Cruz, SC-4327	1:100	Microwave	C, M	62	C, M
X71	p-PDGFR-β (Tyr1021)	Santa Cruz, SC-12909-R	1:100	Microwave	M	62	C, M
X72	VEGF	Santa Cruz, SC-152	1:300	Microwave	C, M	62	C
X73	VEGFR2	Cell Signaling, 2479	1:125	Microwave	C, M	62	C, M
X74	p-VEGFR2 (Tyr1175)	Cell Signaling, 2478	1:300	Microwave	C, M	62	C, M
X75	p-VEGFR2/3 [GLARDIpYKDPDpYVRKGD(C)]	Calbiochem, PC460	1:2000	Microwave	C, M	62	C, M
X76	EGF	Santa Cruz, SC-275	1:25	Microwave	C, M	62	C, M, N
X77	EGFR	Santa Cruz, SC-03	1:50	Microwave	M	62	C, M
X78	p-EGFR (Tyr1173)	Biosource, 44–794G	1:50	Microwave	C, M	62	C, M
X79	MMP2	Chemicon, ab807	1:200	Microwave	C, M	62	C, M
X80	MMP9	Chemicon, ab13458	1:100	Microwave	C, M	62	C
X81	Cleaved caspase-3	Cell Signaling, 9661	1:200	Microwave	C, N	62	C, N
X82	Wile-type EGFR	Dako, M7298	1:1000	Microwave	M	62	C, M
X83	EGFR vIII	Abcam, ab52104	1:50	Microwave	C, M	62	C, M
X84	Notch	Abcam, ab27526	1:200	Microwave	C, M, N	62	C, M, N

C, cytoplasm; M, membrane; N, nucleus; APC, *Adenomatous polyposis coli*; CDC25B, cell division cycle 25 homolog B; DCC, deleted in colorectal carcinoma; Dnmt1, DNA methyltransferases 1; E2F-1, E2F transcription factor 1; EGF, epidermal growth factor; EGFR, epidermal growth factor receptor; FHIT, fragile histidine triad; Id4, inhibitor of differentiation 4; MDM2, murine double minute-2; MIB-1, mindbomb homolog 1 (*Drosophila*); MLH1, mutL homolog 1; MMP, matrix metallopeptidase; PDGF, platelet-derived growth factor; PDGFR, platelet-derived growth factor receptor; Pgp, P-glycoprotein; PTEN, phosphatase and tensin homolog; RAR-β, retinoic acid receptor; Rb, retinoblastoma protein; SMA, α-smooth muscle actin; Sox-2, sex determining region Y-box 2; TGF, transforming growth factor; TIMP-3, tissue inhibitor of metalloproteinase; VEGF, vascular endothelial growth factor; VEGFR1, vascular endothelial growth factor receptor 1.

**Table II:** Clinical characteristics of patients from the original set.

Characteristics	Cluster 1 (no.)	Cluster 2 (no.)
Patients	19	37
Gender (male:female)	11:8	23:14
Mean age (years)	51.6	55.6
Pathologic subtype (primary:secondary)	18:1	36:1
Surgical treatment		
Total resection (%)	12 (63.2%)	29 (78.4%)
Partial resection (%)	7 (36.8%)	7 (18.9%)
Biopsy (%)	0 (0.00%)	1 (2.70%)
RT + temozolomide (%)	15 (78.9%)	29 (78.4%)

**Table III:** Cox proportional hazards multivariate analysis in overall survival.

Variable	Hazard ratio (95% CI)	p-value
Molecular classification(cluster 1 vs. cluster 2)	0.342 (0.154–0.7570)	<0.01
Age (<70 years vs. ≥ 70 years)	4.728 (1.683–13.286)	<0.01
Karnofsky performance status (KPS) (<70 vs. ≥ 70)	2.496 (0.593–10.495)	0.21
University of California San Francisco (UCSF) grade^a^	0.641 (0.444–0.9250)	0.018
Extent resection	0.340 (0.097–1.1930)	0.092

a The UCSF grade was determined by the spatial relationship of the contrast-enhancing lesion (CEL) with the subventricular zone (SVZ) and cortex (46).

Classification was as follows: group I, CEL contacting SVZ and infiltrating cortex; group II, CEL contacting SVZ but not involving cortex; group III, CEL not contacting SVZ but involving cortex; and group IV, CEL neither contacting SVZ nor infiltrating cortex.

**Table IV:** Adjusted p-value for Student’s t-test for each protein and cellular location pairs.

Code	Protein	Localization	Adjusted p-value	Up-/down-regulated at chemo-radiation-resistant group
X50	Cyclin E	Nuclear	0.00744	Up
X16	DCC	Nuclear	0.01550	Up
X41	Survivin	Nuclear	0.02950	Up
X44	TGF-β	Cytoplasm	0.01040	Up
X08	CDC25B	Nuclear	0.00744	Down
X24	Histone H1 (B419)	Nuclear	0.01130	Down
X78	p-EGFR	Cytoplasm	0.02950	Down
X75	p-VEGFR2/3	Cytoplasm	0.01220	Down
X31	p16	Cytoplasm	0.00744	Down
X31	p16	Nuclear	0.00744	Down

**Table V:** Clinical characteristics of patients from the validation set.

Characteristics	Cluster 1A (no.)	Cluster 2B (no.)
Patients	59	23
Gender (male:female)	35:24	15:8
Mean age (years)	53.9	54.2
Pathologic subtype (primary:secondary)	56:3	21:2
Surgical treatment		
Total resection (%)	40 (67.8%)	19 (82.6%)
Partial resection (%)	15 (25.4%)	4 (17.4%)
Biopsy (%)	4 (6.80%)	0 (0.00%)
RT + temozolomide (%)	46 (77.9%)	19 (82.6%)
